# Exercise and Omega-3 Polyunsaturated Fatty Acid Supplementation for the Treatment of Hepatic Steatosis in Hyperphagic OLETF Rats

**DOI:** 10.1155/2012/268680

**Published:** 2011-09-12

**Authors:** Sarah J. Borengasser, R. Scott Rector, Grace M. Uptergrove, E. Matthew Morris, James W. Perfield, Frank W. Booth, Kevin L. Fritsche, Jamal A. Ibdah, John P. Thyfault

**Affiliations:** ^1^Departments of Nutrition and Exercise Physiology, University of Missouri, Columbia, MO 65201, USA; ^2^Division of Animal Sciences, University of Missouri, Columbia, MO 65201, USA; ^3^Internal Medicine-Division of Gastroenterology, University of Missouri, Columbia, MO 65201, USA; ^4^Harry S Truman Memorial Veterans Hospital, Research Service, Columbia, MO 65201, USA; ^5^Food Science-Division of Food Systems & Bioengineering, University of Missouri, Columbia, MO 65201, USA; ^6^Department of Biomedical Sciences, University of Missouri, Columbia, MO 65201, USA; ^7^Department of Medical Pharmacology and Physiology, University of Missouri, Columbia, MO 65201, USA; ^8^Dalton Cardiovascular Center, University of Missouri, Columbia, MO 65201, USA

## Abstract

*Background and Aims.* This study examined if exercise and omega-3 fatty acid (n3PUFA) supplementation is an effective treatment for hepatic steatosis in obese, hyperphagic Otsuka Long-Evans Tokushima Fatty (OLETF) rats. *Methods.* Male OLETF rats were divided into 4 groups (*n* = 8/group): (1) remained sedentary (SED), (2) access to running wheels; (EX) (3) a diet supplemented with 3% of energy from fish oil (n3PUFA-SED); and (4) n3PUFA supplementation plus EX (n3PUFA+EX). The 8 week treatments began at 13 weeks, when hepatic steatosis is present in OLETF-SED rats. *Results.* EX alone lowered hepatic triglyceride (TAG) while, in contrast, n3PUFAs failed to lower hepatic TAG and blunted the ability of EX to decrease hepatic TAG levels in n3PUFAs+EX. Insulin sensitivity was improved in EX animals, to a lesser extent in n3PUFA+EX rats, and did not differ between n3PUFA-SED and SED rats. Only the EX group displayed higher complete hepatic fatty acid oxidation (FAO) to CO_2_ and carnitine palmitoyl transferase-1 activity. EX also lowered hepatic fatty acid synthase protein while both EX and n3PUFA+EX decreased stearoyl CoA desaturase-1 protein. *Conclusions.* Exercise lowers hepatic steatosis through increased complete hepatic FAO, insulin sensitivity, and reduced expression of *de novo* fatty acid synthesis proteins while n3PUFAs had no effect.

## 1. Introduction

It is estimated that 20–40% of the general population and 50–90% of obese people have nonalcoholic fatty liver disease (NAFLD) [1, 2]. The presence of NAFLD has been described as an independent risk factor for the development of cardiovascular disease [3–5], and it often accompanies other obesity-related diseases such as type 2 diabetes and is considered the hepatic manifestation of the metabolic syndrome [6]. Further, NAFLD is associated with increases in all-cause and liver-related mortality [7, 8]. As a result, the examination of affordable treatment options for NAFLD, such as exercise or omega-3 polyunsaturated fatty acid (n3PUFA) supplementation, is becoming more imperative. Unfortunately, there is a paucity of studies investigating exercise and exercise combined with n3PUFA supplementation as treatments for NAFLD. Most exercise research has been focused on prevention, and not on treatment or reversal of preexisting NAFLD. 

We have previously used the Otsuka Long-Evans Tokushima Fatty (OLETF) rats to investigate NAFLD because they spontaneously develop obesity, insulin resistance, and NAFLD which progressively worsens with advancing age [9, 10]. OLETF rats lack the cholecystokinin-1 receptor resulting in hyperphagia due to defective satiety signaling [11]. Further, a novelty of this particular model is their intrinsic aptitude for exercise (EX) on voluntary running wheels, a characteristic that is uncommon in obese rodent models. We have previously shown that EX on voluntary wheel running beginning at 4 weeks of age in OLETF rats prevents the development of hepatic steatosis, effects that were associated with prevention of insulin resistance and increased hepatic fatty acid oxidation (FAO) [9]. Moreover, we have established that locking of the wheels and ceasing daily EX in OLETF rats quickly activate a subgroup of precursors and processes known to initiate hepatic steatosis, including decreased hepatic mitochondrial oxidative capacity, increased hepatic expression of *de novo *lipogenesis proteins, and increased hepatic malonyl CoA levels [12]. Other labs have also shown that exercise can prevent fatty liver in rodents fed a high-fat-diet to induce obesity [13, 14]. Although we and others have established that daily exercise is an invaluable intervention to prevent NAFLD, it remains unknown if exercise is an effective treatment strategy for preexisting NAFLD.

Dietary polyunsaturated fatty acids (n3PUFAs) have also been studied as a treatment for NAFLD. n3PUFAs both activate a transcription factor that is important for hepatic oxidative capacity (peroxisome proliferator-activated receptor (PPAR*α*)) and inactivates a master regulator of hepatic lipid synthesis (sterol regulatory element binding protein (SREBP)-1c) in rodents [15–22] and humans [23, 24]. This dual action on hepatic metabolism suggests that n3PUFAs could lower hepatic fat content. In OLETF rats, dietary intake of n3PUFAs beginning at 5 weeks of age prevented hepatic steatosis by decreasing the activity of lipogenic enzymes such as fatty acid synthase (FAS) and stearoyl CoA desaturase (SCD)-1 and decreasing mRNA expression of several key lipogenic genes including SREBP-1c and acetyl CoA carboxylase (ACC) [25]. The authors also found that mRNA expression and activity of CPT, a protein that is controlled by PPAR*α*, were increased in the liver of n3PUFA-fed OLETFs [25]. These results indicate that n3PUFAs were effective at preventing increased liver TAG accumulation in OLETF rats; however, this study also addressed prevention and not treatment of NAFLD.

Treatment of NAFLD with n3PUFAs has also been examined previously. Seven days of dietary consumption of n3PUFAs led to reductions, but not complete reversal, of hepatic steatosis, and systemic insulin resistance in *ob/ob* mice, associated with the down-regulation of SREBP-1c [19]. The authors also observed increased activation of PPAR*α* and its associated gene targets suggesting increased *β*-oxidation in the liver, but lacked a quantitative measure of FAO. In another study, dietary intake of n3PUFAs attenuated, but was again not able to completely reverse, liver TAG content following high-fat-diet-induced hepatic steatosis [20]. 

To our knowledge, we are unaware of any studies that have examined the individual and combined effects of exercise and n3PUFA supplementation on hepatic mitochondrial FAO and treatment of NAFLD. Thus, the purpose of this study was to examine the individual and combined effects of exercise and the dietary intake of omega-3 fatty acids (3% of energy from fish oil) to treat preexisting hepatic steatosis in OLETF rats. We also examined the effects of these treatments on factors known to play a role in the development of hepatic steatosis including peripheral insulin sensitivity, hepatic FAO, and markers of hepatic lipogenesis. We hypothesized that both exercise and n3PUFAs would reverse or attenuate progression of NAFLD in obese rats and that the combination of treatments would have an even greater effect on treating NAFLD. 

## 2. Methods

### 2.1. Study Design

 The animal protocol was approved by the Institutional Animal Care and Use Committee at the University of Missouri. Male OLETF rats were generously provided by the Tokushima Research Institute, Otsuka Pharmaceutical (Tokushima, Japan) and arrived at 4 weeks of age. Rats were given *ad libitum* access to normal chow food (Purina Formulab Diet, 5008) and water in a temperature- and light-controlled room (12 h light-12 h dark cycle). At 13 weeks of age, an age when obesity, insulin resistance, and NAFLD are present in sedentary OLETF rats [10], OLETFs were randomly divided into 4 groups (*n* = 8/group): (1) remained sedentary (SED) on normal chow, (2) was provided access to voluntary running wheels (EX) with normal chow, (3) was provided diet supplemented with 3% of energy from fish oil (n3PUFA-SED), and (4) had access to running wheels and diet supplemented with 3% of energy from fish oil (n3PUFA+EX). Thus, treatments began at 13 weeks of age and ended at 20 weeks of age when rats were euthanized. Food consumption and body weights were assessed weekly, and running distances (wheels outfitted with a Sigma Sport BC 800 bicycle computer (Cherry Creek Cyclery, Foster Falls, VA, USA) for measuring daily running activity) in EX and n3PUFA-EX rats were measured daily following the end of the dark cycle during the 8-week treatment period. After being fasted and having wheels locked for a 5 h period, rats were anesthetized with sodium pentobarbital (100 mg/kg), tissue and blood were collected, fat pads were weighed, and rats were euthanized by exsanguination. 

### 2.2. Diet

 The normal chow diet used was Formulab 5008 (Purina Mills, St. Louis, Mo, USA) which we have used in previous studies in which the OLETF rats develop steatosis [9, 10]. The n3PUFA diet was custom-formulated at the University of Missouri using an AIN-93G diet supplemented with 3% of energy from menhaden fish oil. The menhaden fish oil was kindly donated by Omega Protein, Inc. (Houston, Tex, USA) and was stabilized with a synthetic antioxidant (0.2 g/kg tertiary butyl-hydroquinone (TBHQ)) and 1000 mg/kg mixed tocopherols to protect it from autooxidation. The percent of energy provided by fat was the same between the Formulab 5008 and n3PUFA diet, 16.7%, respectively.

### 2.3. Intraperitoneal Glucose Tolerance Tests

Baseline glucose values were determined following a 12 h overnight fast one week prior to sacrifice (19 weeks of age). Glucose (2.0 g/kg) was then administered intraperitoneally, and tail blood draws were taken prior to and 15, 30, 45, 60, and 120 min following the bolus of glucose as performed previously by our group [26].

### 2.4. Dual-Energy X-Ray Absorptiometry (DEXA)

 Whole body composition was determined using a Hologic QDR-1000/w DEXA machine calibrated for rats as performed previously by our group [27]. 

### 2.5. Serum Measures

 The concentrations of glucose (Sigma Aldrich, St. Louis, Mo, USA), insulin (Millipore, Billerica, Mass, USA), *β*-hydroxybutyrate (Stanbio Laboratory, Boerne, Tex, USA), triglycerides (Sigma Aldrich), and free fatty acids (Wako, Richmond, Va, USA) in plasma were measured using commercially available kits. 

### 2.6. Liver Tissue Procedures

Livers were quickly excised from anesthetized rats and were either immediately freeze-clamped in liquid nitrogen, placed in 10% formalin, or placed in ice-cold isolation buffer and then homogenized as previously described [9]. 

### 2.7. Fatty Acid Oxidation

 Fresh tissue fatty acid oxidation (FAO) using radiolabeled ^14^C palmitate (Perkin Elmer, Boston, Mass, USA) was performed as previously described [27]. Briefly, both ^14^CO_2_, representing complete fatty acid oxidation, and ^14^C-labeled acid soluble metabolites (^14^C ASM), representing incomplete fatty acid oxidation, were collected in the previously described trapping device and then counted on a liquid scintillation counter. In addition to the ^14^C palmitate, the reaction buffer contained a final concentration of 50 *μ*M cold palmitate and 0.5% bovine serum albumin.

### 2.8. Enzyme Assays

 Carnitine palmitoyl transferase-1 (CPT-1) activity, *β*-hydroxy-acyl-CoA dehydrogenase activity (*β*-HAD), and citrate synthase activity assays were performed as described previously by our group [27]. 

### 2.9. Hepatic Nuclear Isolation

 Nuclear extraction of fresh liver tissue was performed using a commercially available kit (Marligen Biosciences, Ijamsville, Md, USA). Briefly, fresh tissue was immediately placed into hypotonic lysis buffer, homogenized, washed, vortexed, and centrifuged. Supernatant containing the cytoplasmic extracts was removed. Each sample was washed by centrifugation until the supernatant was clear. Samples were incubated on ice followed by a 30 min high-speed centrifugation at 4°C. Supernatant was removed representing the nuclear extract and stored at −80°C. 

### 2.10. Hepatic TAG and DAG Analysis

Hepatic fatty acids were extracted using a modified Folch method [28]. Briefly, liver tissue was homogenized in ice-cold Trizma/EDTA buffer and lipids were extracted in chloroform/methanol/acetic acid (2 : 1 : 0.15). Extracted lipids were then run on a thin-layer chromatography (TLC) silica plate in a tank containing hexane, diethyl ether, and acetic acid (70 : 30 : 1). Triglyceride and diacylglycerol fractions were scraped from the TLC plate and were then methylated by incubating with toluene, methanol, and acetyl chloride (0.5 : 1.2 : 0.1) at 100°C for 60 min and separated in hexane. Fatty acid methyl esters were analyzed by gas chromatography (Agilent Technologies, Wilmington, De, USA). 

### 2.11. Liver Histology

Hematoxylin and eosin (H&E) staining was performed on formalin-fixed paraffin-embedded section of liver as described previously to assess lipid vacuolization [29].

### 2.12. Western Blotting

 Western blots were performed to detect protein levels of PPAR*α* (Santa Cruz Biotechnology, Santa Cruz, Calif, USA), PPAR*δ* (Thermo Scientific, Rockford, Il, USA), PPAR*γ* (Cell Signaling, Beverly, CA), SREBP-1c (Santa Cruz Biotechnology), fatty acid synthase (FAS) (Cell Signaling), acetyl coenzyme A carboxylase (ACC) (Cell Signaling), and stearoyl CoA desaturase (SCD)-1 (Alpha Diagnostic International, San Antonio, Tex, USA) as performed previously [9]. Liver samples were homogenized in ice-cold lysis buffer, separated by SDS-PAGE gels, transferred to PVDF membranes, and probed with primary antibodies. Protein bands were quantified using a densitometer and band densities were corrected for total protein loaded by staining with 0.1% amido black (Sigma) as described previously [9].

### 2.13. Gene Expression

 Quantitative RT-PCR was performed as previously described for mRNA expression of PPAR*α* and PPAR*δ* [26]. Briefly, on the day of sacrifice, fresh tissue was collected and placed in RNA*later* (Ambion, Austin, Tex, USA) stored at 4°C for 24 h and then stored at −20°C. Liver samples were pulverized in RLT buffer using the QIAGEN TissueLyser system (Valencia, Calif, USA). RNA was then isolated using a commercially available kit (QIAGEN, Valencia, Calif, USA). Reverse transcription and cDNA synthesis were performed according to the manufacturer instructions (Promega, Madison, Wis, USA). TaqMan Master Mix (Applied Biosystems, Foster City, Calif, USA) for gene targets PPAR*α* and PPAR*δ* were loaded into a 96-well microplate along with the cDNA sample (5 *μ*g/*μ*L) and placed into the ABI 7000 Sequence Detection System for polymerization. Results were quantified relative to *β*-actin. Critical threshold (Ct) values for the housekeeping gene did not differ among groups (data not shown).

### 2.14. Statistical Analysis

 Treatment differences were analyzed by one-way analysis of variance (ANOVA) with main effect significance set at *P* < 0.05. Significant main effects were then followed up by least significant difference (LSD) post hoc comparisons (SPSS 17.0). Data are presented as means ± SE, and significance was set at *P* < 0.05. 

## 3. Results

### 3.1. Animal and Serum Characteristics

 Final body weight and fat pad mass (omental + retroperitoneal) were significantly different among all groups (Table 1). Body fat percentage was highest in the SED and n3PUFA-SED rats; EX rats had the lowest body fat percentage, over 2.5fold lower than SED and n3PUFA-SED and 1.6fold lower than n3PUFA+EX. Fat pad mass mirrored the body fat percentage measures except that n3PUFA-SED was lower than SED. Weekly food intake (grams) was reduced in the EX and n3PUFA+EX groups as compared to SED and n3PUFA-SED. However, when weekly food intake (grams) was normalized to body weight, the EX group consumed more than the SED, n3PUFA-SED, and n3PUFA+EX groups. Weekly caloric intake per body weight was also highest in the EX compared to all other groups. In addition, the SED group consumed more Kcal per body weight than the two groups who received n3PUFA supplementation. All 3 treatment conditions significantly lowered circulating TAG and FFAs compared with SED rats.

### 3.2. Weekly Run Distance and Body Weight

**Figure 1:**
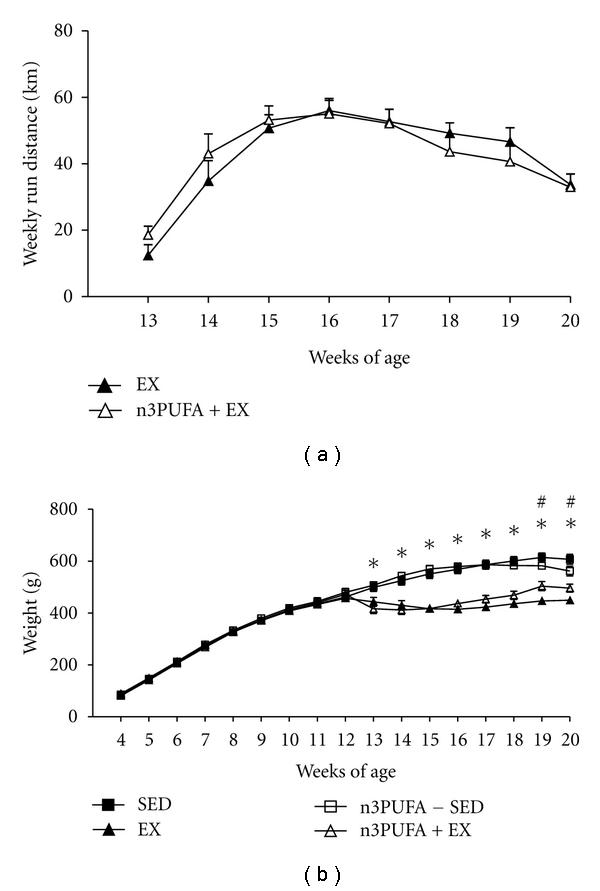
Running distance and body weights. Weekly run distance (a) during the 8 week treatment intervention and weekly body weight (b). Values are means ± SE (*n* = 8). *EX and n3PUFA+EX are significantly different from SED and n3PUFA-SED (*P* < 0.05). ^#^EX is significantly different from all groups, and n3PUFA+EX is significantly different than SED and n3PUFA-SED.

 EX and n3PUFA+EX rats were given access to running wheels at 13 weeks of age. There were no differences in weekly run distances between the two groups (Figure 1(a)). Peak running distances in both groups were approximately 56 and 55 km per week (approximately 8 km per night) at 16 weeks and slowly declined to about 34 and 33 km per week (*∼*5 km per night) by 20 weeks of age. As shown in Figure 1(b), EX and n3PUFA+EX rats showed a decline in body weight the first two weeks following introduction to wheel running and then began gaining weight again while SED and n3PUFA-SED rats continued to gain weight during the 8 weeks of treatment. 

### 3.3. Hepatic Steatosis

**Figure 2:**
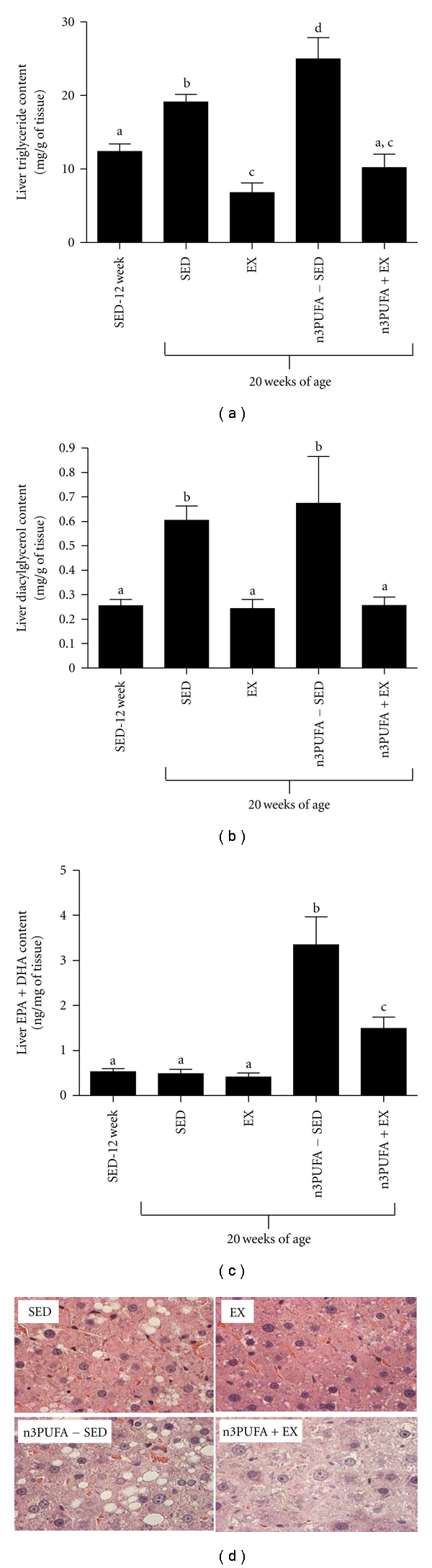
Hepatic fat storage. Fat accumulation in the liver as shown by liver triglyceride content (a), liver diacylglycerol content (b), liver n3PUFA content (EPA + DHA) (c), and representative images of hematoxylin and eosin staining for lipid vacuolization (d). Values are means ± SE (*n* = 8). Values with different letter superscripts are significantly different from each other (*P* < 0.05).

Hepatic TAG content from previously studied 13-week-old OLETF-SED rats (*n* = 8) was used as a baseline measure of hepatic steatosis allowing us to determine if the 8-week treatments could reverse steatosis. As shown in Figure 2(a), EX rats had lower TAG content at 20 weeks of age as compared to the baseline TAG content of SED-12 wk rats (*P* = 0.03), indicating EX effectively treated NAFLD. Although the beneficial effects of EX were partially blunted in the n3PUFA+EX animals, liver TAG content was significantly lower than SED-20 wk (*P* = 0.001). Unexpectedly, n3PUFA supplementation in sedentary, hyperphagic rats exacerbated hepatic steatosis above that measured in both SED groups (*P* < 0.05; Figure 2). Hepatic DAG content (Figure 2(b)) followed similar trends as TAG content, with EX and n3PUFA+EX both offering protection against the accumulation of DAGs in the liver as compared to SED and n3PUFA-SED. The n3PUFA content eicosapentaenoic acid (EPA) and docosahexaenoic acid (DHA) were measured in the liver and showed that EPA and DHA were incorporated into the liver at a higher level in n3PUFA-fed rats with n3PUFA-SED having the highest content (Figure 2(c)). Representative images of H&E staining for lipid vacuolization (Figure 2(c)) demonstrate a visual presence of steatosis in n3PUFA-SED and SED rats as shown by the large size and number of lipid droplets, whereas EX and n3PUFA+EX displayed noticeably less lipid droplets.

### 3.4. Systemic Insulin Sensitivity

**Figure 3:**
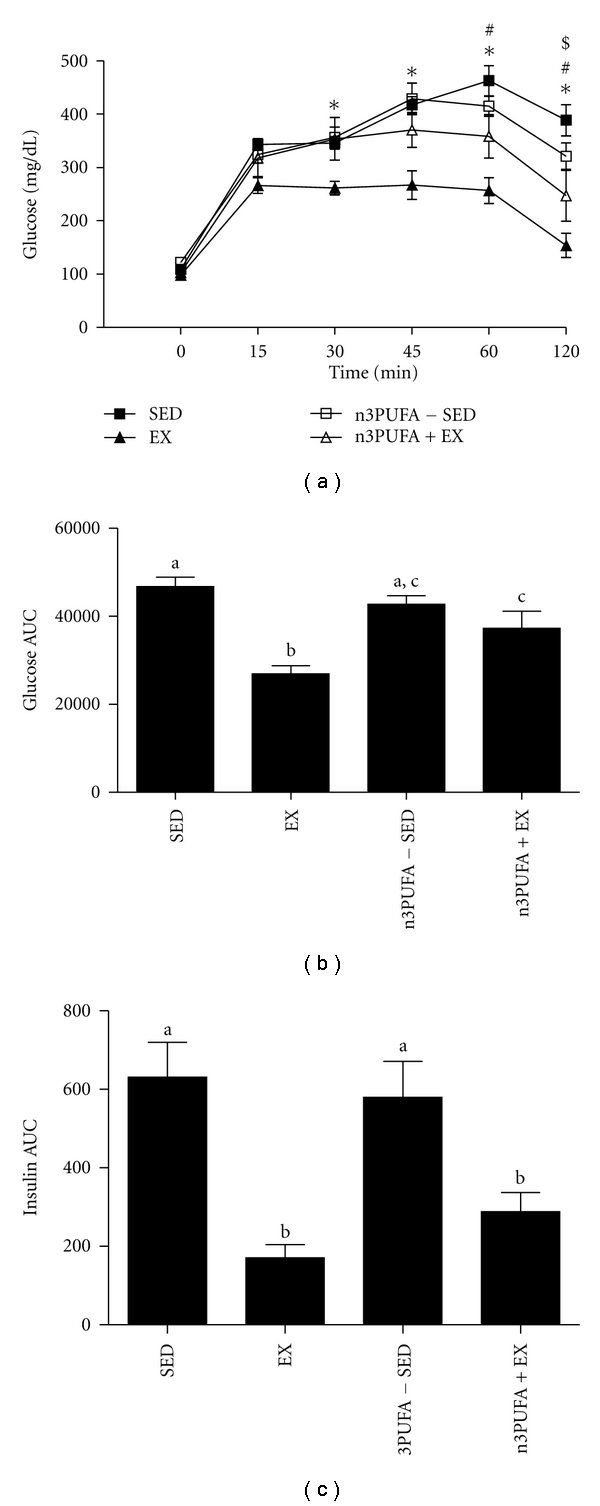
Insulin sensitivity. Peripheral insulin sensitivity was assessed by intraperitoneal glucose tolerance tests represented as changes in blood glucose over time (a), glucose area under the curve (AUC) (b), and insulin AUC (c). Values are means ± SE (*n* = 8). *EX is different from other groups, ^#^n3PUFA+EX is different from SED, ^$^n3PUFA+EX is different from n3PUFA-SED (*P* < 0.05). Values with different letter superscripts are significantly different from each other (*P* < 0.05).

 Blood glucose and insulin levels were elevated in SED rats as compared to EX, n3PUFA-SED, and n3PUFA+EX (Table 1). Glucose responses to the tolerance test are shown in Figure 3(a). Glucose and insulin AUCs during the IPGTT did not differ between n3PUFA and SED groups, whereas EX rats had significantly lower glucose and insulin AUCs (*P* < 0.01), and n3PUFA-EX rats only had lower insulin AUCs (*P* < 0.05) as shown in Figures 3(b) and 3(c). 

### 3.5. Hepatic Mitochondrial Function and Fatty Acid Oxidation

**Figure 4:**
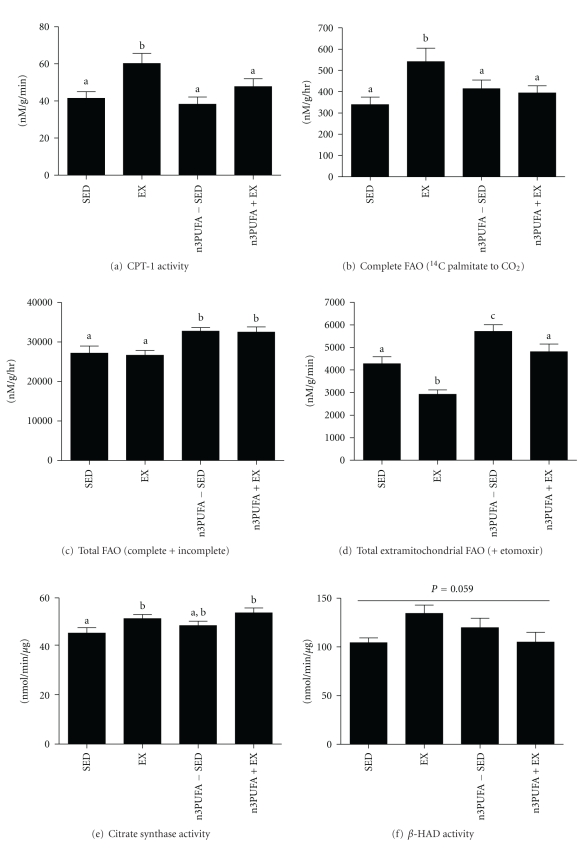
Hepatic fatty acid oxidation. Carnitine palmitoyl transferase (CPT)-1 activity (a), hepatic complete fatty acid oxidation (^14^CO_2_) (b), hepatic total fatty acid oxidation (^14^CO_2_ + ^14^C ASM) (c), hepatic total extramitochondrial fatty acid oxidation (^14^CO_2_ + ^14^C ASM) in the presence of etomoxir (d), citrate synthase activity (e), and *β*-HAD activity (f). Values with different letter superscripts are significantly different from each other (*P* < 0.05).

Only the EX group had higher CPT-1 activity than SED (Figure 4(a); *P* < 0.05). EX and n3PUFA+EX had higher citrate synthase activity followed by SED (Figure 4(e)), and there were no differences among groups in *β*-HAD activity, though a main effect of treatment groups versus SED approached significance (Figure 4(f); *P* = 0.059). Rats in the n3PUFA-SED and n3PUFA+EX groups had significantly higher total FAO (^14^CO_2_ + ^14^C ASM) as compared to SED and EX rats (Figure 4(c)). This elevation in total FAO was due to elevated incomplete fatty acid oxidation as shown by higher ASM production (data not shown). Further, in the presence of etomoxir, which inhibits CPT-1 thereby ceasing the transport of long-chain fatty acids to the mitochondria, n3PUFA-SED and n3PUFA+EX had higher rates of FAO than the EX group but not the SED group (Figure  4(d)). This suggests that the dietary intake of n3PUFAs increases extramitochondrial total FAO in both SED and EX conditions. In addition, EX was the only group to have significantly higher complete FAO (^14^CO_2_) as compared to SED, n3PUFA-SED, and n3PUFA+EX as shown in Figure 4(b). 

### 3.6. Hepatic Transcription Factors and Fatty Acid Synthesis Pathway

**Figure 5:**
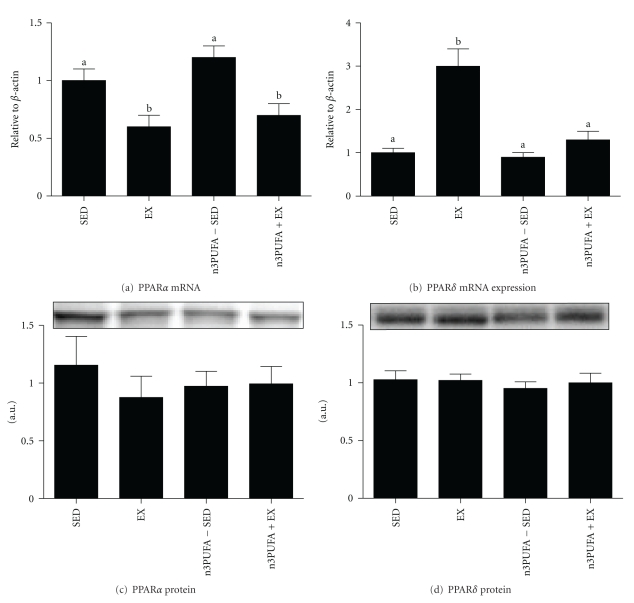
PPAR expression. mRNA expression of PPAR*α* (a) and PPAR*δ* (b) and protein content of PPAR*α* (c) and PPAR*δ* (d). Values with different letter superscripts are significantly different from each other (*P* < 0.05).

**Figure 6:**
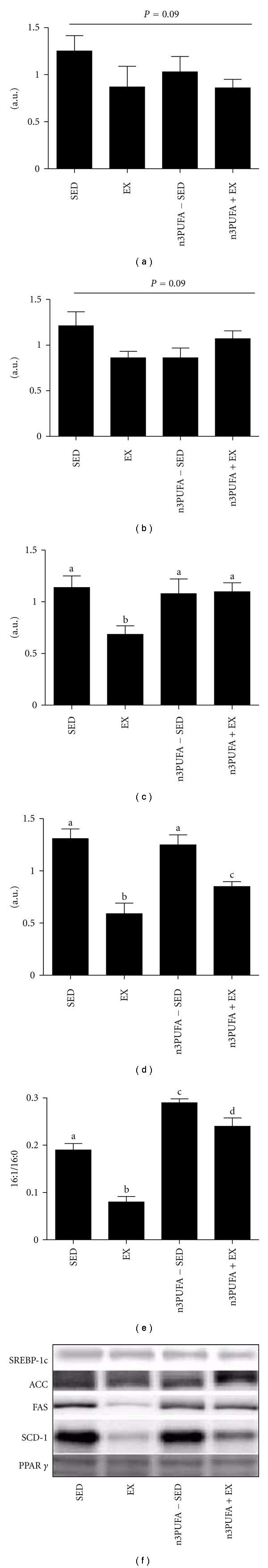
Lipogenic markers. Protein content of nuclear SREBP-1c (a), ACC (b), FAS (c), SCD-1, and PPAR*γ* (d). Representative blots are shown in (f) for each of the target proteins. Desaturase index is indicative of SCD-1 desaturase activity and is shown by the ratio of 16 : 1 to 16 : 0 (e). Values with different letter superscripts are significantly different from each other (*P* < 0.05).

PPAR*α* mRNA expression was significantly lower in EX and n3PUFA+EX (Figure 5(a)). The expression of PPAR*δ*, a transcription factor associated with mitochondrial biogenesis and fatty acid oxidation, was 3-fold higher in EX as compared to SED, n3PUFA-SED, and n3PUFA+EX (Figure 5(b)). However, there were no differences in protein expression of PPAR*α* or *δ* among groups as shown in Figures 5(c) and 5(d). The nuclear protein content of SREBP-1c did not differ among groups, although there was a trend toward significance (*P* = 0.09) as seen in Figure 6(a). There also was a trend (*P* = 0.09) for a main effect for treatment on ACC protein content. However, the EX rats had significantly lower FAS protein content (*P* = 0.02) as compared to SED, n3PUFA-SED, and n3PUFA+EX. There were no differences in PPAR*γ* protein content among groups (representative western blot shown in Figure 6(f)). EX rats also had the lowest protein content of SCD-1 as compared to the other groups (Figure 6(d)). SCD-1 desaturase index was lowest in EX and highest in n3PUFA-SED rats as estimated by product (16 : 1) to substrate (16 : 0) ratio for the enzyme (Figure 6(e)). 

## 4. Discussion

The primary objectives of the current study were to determine if exercise and n3PUFA supplementation, alone or in combination, could effectively treat hepatic steatosis in obese OLETF rats and to determine if these treatment strategies were associated with changes in insulin sensitivity, hepatic mitochondrial FAO, or markers of hepatic lipogenesis. The major findings include the following: (1) exercise treatment reversed preexisting hepatic steatosis in association with elevated hepatic complete FAO and CPT-1 activity, improved systemic insulin sensitivity, and decreased markers of hepatic lipogenesis; (2) n3PUFAs supplemented at 3% of diet increased TAG accumulation in the liver and, when combined with exercise n3PUFAs, did not lead to additive health benefits, but instead partially blunted the beneficial effects of exercise to reduce hepatic TAG accumulation and improve insulin sensitivity.

EX was the only treatment that reversed liver TAG content below the pretreatment levels measured in 13-week-old sedentary OLETF animals. It is likely that EX lowered hepatic steatosis in part by slowing gains in body weight and fat mass that normally occur in SED conditions. However, the data also supports the notion that EX induced a hepatic metabolic phenotype that played a role in reducing steatosis as well. Evidence suggests that EX increased both the mitochondrial entry rate (higher CPT-1 activity (Figure 4(a)) and complete catabolism of long-chain fatty acids (increased complete palmitate oxidation to CO_2_ (Figure 4(b)). The increase in complete oxidation is likely due to a more efficient coupling of increased *β*-oxidation and TCA cycle flux. EX also lowered extramitochondrial hepatic FAO (oxidation that occurs in the presence of CPT-1 inhibition with etomoxir (Figure 4(d)). Our assumption is that extramitochondrial oxidation is primarily driven by peroxisomes, organelles that break down longer chain fatty acids so that they can then be oxidized in the mitochondria. Peroxisomal activity has previously been shown to be higher in the SED OLETF [30] and when not paired with increased mitochondrial FAO [31–33] could lead to the accumulation of fatty acid metabolites and oxidative stress. 

Livers were examined 5 hours after the last exercise bout, opening the possibility that the exercise-induced adaptations were primarily due to the last bout of exercise rather than a result of chronic training. In a previous study from our group, OLETF rats exercised on voluntary running wheels for XX weeks before we locked the wheels to stop exercise [12]. We then examined hepatic responses 5 hours, 53 hours (2 days), and 173 hours (7 days) after the last exercise bout allowing us to determine if 2 or 7 days of inactivity led to different outcomes than the group studied 5 hours after the last bout. In addition, all 3 groups were also compared to OLETFs who were never provided access to running wheels. Most measures of mitochondrial content were unchanged by 7 days of inactivity and were still higher than sedentary livers, while markers of *de novo* lipogenesis (FAS and ACC protein content and malonyl CoA) increased significantly 2 and 7 days after the cessation of exercise. Hepatic TAG did not increase significantly 7 days after the cessation of exercise and remained significantly lower than the content measured in sedentary rats. Thus, these data suggest that some hepatic adaptations to EX treatment are sustained and due to chronic training while others are dependent on the effects of the last bout.

Peripheral insulin resistance is strongly associated with hepatic steatosis. Importantly, high levels of circulating insulin activate SREBP-1c, a master regulator of lipid synthesis. Here we report that EX had the most powerful effects on reducing glucose and insulin levels during the IPGTT. Similar to our previous reports of exercise preventing the development of insulin resistance in the OLETF model [9, 12, 34], here we demonstrate that exercise also can be used as a treatment to improve insulin sensitivity. We also originally hypothesized that EX or dietary n3PUFAs would reduce the mature form of SREBP-1c and would play a key role in downregulating fatty acid synthesis in the liver [16, 18, 35–37]. In addition, we hypothesized that n3PUFAs would activate PPAR*α* and *δ*, as n3PUFAs are known to be natural ligands and potent activators of PPARs. Surprisingly, the mature, nuclear form of SREBP-1c was not significantly altered by any of the treatments and n3PUFAs did not potently increase expression or protein content of PPARs. However, although there were no changes in SREBP-1c, protein content of FAS and SCD-1, its downstream targets, was dramatically reduced with EX. It is possible that the trend for lowering of nuclear SREBP-1c was enough to lower FAS and SCD-1 or that EX lowered these factors through other pathways. Through the conversion of saturated fatty acids to monounsaturated fatty acids, hepatic SCD-1 contributes to abnormal partitioning of fatty acids by reducing FAO and increasing fatty acid synthesis. EX significantly suppressed SCD-1 expression and activity, resulting in lower desaturation of the fatty acid within hepatic TAG which is consistent with our previous results. In contrast, n3PUFA did not impact SCD-1 content and slightly increased activity as shown by the desaturase index. FAS is also a critical step in the conversion of carbohydrates to fatty acids through *de novo* lipogenesis. All told, EX lowered markers of hepatic lipogenesis, but because this occurred without a clear lowering of nuclear SREBP-1c content, we are unsure these changes are directly linked to reduced circulating insulin and improved systemic insulin sensitivity. 

Contrary to our original hypothesis and the results of others, we found that n3PUFA supplementation in SED OLETFs led to increased TAG accumulation in the liver [16, 19, 20, 35, 38]. Although our results are conflicting with a large majority of the literature, it should be noted that the majority of fish oil/n3PUFA research conducted in animals uses excessive amounts (6–60% of energy) that are not likely physiologically relevant [16, 35–41]. Here we used a diet consisting of 3% of total energy from fish oil which is a concentration that can safely be consumed by humans [42]. Consequently, it may require higher dosages of n3PUFAs to yield the health benefits found by others in their rodent models. In addition, we started treatment at 13 weeks of age, an age where OLETFs several obesity-induced comorbidities such as NAFLD, systemic insulin resistance, and increased adiposity are present which may explain the differences seen between prevention and treatment with n3PUFAs. An alternative and more probable explanation is that dietary consumption of “healthy” fatty acids cannot likely overcome a hyperphagic, chronic state of positive energy balance and daily weight gain in sedentary rats. Excess energy intake, regardless of the source, will likely result in increased adiposity and ectopic fat storage if not matched with increased energy expenditure. Rather, either lowering total energy intake or increasing energy expenditure is likely needed to effectively treat hepatic steatosis. Future experiments are needed to determine if n3PUFA intake would be more effective in reducing steatosis in animals in which the rate of weight gain is reduced by limiting caloric consumption. There are also human clinical implications to these findings. If patients are in a constant positive energy balance and continuing to gain weight, it is unlikely that increasing dietary n3PUFA would lower hepatic fat content. 

In conclusion, our study documents the detrimental effects of overeating while remaining sedentary on adiposity, insulin sensitivity, hepatic mitochondrial function, and hepatic steatosis. Moreover, these adverse events occurred regardless of supplementing n3PUFAs in the diet. However, daily exercise effectively treats hepatic steatosis in obese OLETF rats in part by increasing hepatic mitochondrial function and complete fatty acid oxidation, decreasing activation of the lipid synthesis pathway, and improving systemic insulin sensitivity. Furthermore, treatment with n3PUFAs partially blunted the beneficial effects of exercise in this hyperphagic animal model by unknown mechanisms.

## Figures and Tables

**Table 1:** Animal and serum characteristics.

	SED	EX	n3PUFA-SED	n3PUFA+EX
Final body weight (g)	617.1 ± 20.0^a^	462.0 ± 10.5^b^	571.6 ± 10.3^c^	512.1 ± 17.5^d^
Body fat (%)	31.9 ± 0.9^a^	11.3 ± 1.6^b^	29.5 ± 1.1^a^	18.8 ± 1.3^c^
Fat pad mass (g)	33.1 ± 2.0^a^	9.2 ± 1.6^b^	26.4 ± 1.7^c^	16.4 ± 1.8^d^
Food intake (g)	228.2 ± 7.5^a^	192.2 ± 4.0^b^	226.6 ± 5.8^a^	178.5 ± 3.2^b^
Food wt (g)/BW (g)	0.40 ± 0.00^a^	0.45 ± 0.01^b^	0.40 ± 0.00^a^	0.39 ± 0.01^a^
Caloric intake (kcal)	947.0 ± 31.0^a^	797.8 ± 16.7^b^	853.2 ± 21.9^b^	672.1 ± 11.9^c^
Calories (kcal)/BW (g)	1.67 ± 0.01^a^	1.84 ± 0.05^b^	1.52 ± 0.02^c^	1.48 ± 0.03^c^
Triglycerides (mg/dL)	123.7 ± 17.6^a^	62.1 ± 9.4^b^	59.4 ± 5.4^b^	36.9 ± 4.6^b^
Free fatty acids (*μ*mol/L)	422.6 ± 38.4^a^	199.3 ± 25.8^b^	263.0 ± 25.3^b^	193.4 ± 26.5^b^
Glucose (mg/dL)	396.0 ± 14.1^a^	255.9 ± 16.0^b^	311.9 ± 45.1^b^	239.2 ± 14.5^b^
Insulin (ng/mL)	15.0 ± 1.9^a^	10.0 ± 1.3^b^	11.8 ± 1.6^a,b^	7.6 ± 0.9^b^
Heart wt/body wt (mg/g)	2.5 ± 0.10^a^	3.5 ± 0.01^b^	2.7 ± 0.10^a^	3.1 ± 0.10^b^

Values are means ± SE (*n* = 8). Values with different letter superscripts are significantly different from each other (*P* < 0.05). Fat pad mass: omental + retroperitoneal fat pads; BW: body weight.

## Abbreviations

*β*-HAD: Beta-hydroxyacyl-CoA dehydrogenase
CPT-1: Carnitine palmitoyl-CoA transferase-1
FFAs: Free fatty acids
NAFLD: Nonalcoholic fatty liver disease
OLETF: Otsuka Long-Evans Tokushima Fatty rats
TAG: Triglycerides
n3PUFAs: Polyunsaturated fatty acids.
